# Plasmonic Azobenzene Chemoreporter for Surface-Enhanced Raman Scattering Detection of Biothiols

**DOI:** 10.3390/bios12050267

**Published:** 2022-04-22

**Authors:** Mariacristina Turino, Ramon A. Alvarez-Puebla, Luca Guerrini

**Affiliations:** 1 Department of Physical and Inorganic Chemistry, Universitat Rovira i Virgili, Carrer de Marcel lí Domingo s/n, 43007 Tarragona, Spain; mariacristina.turino@urv.cat; 2 Institució Catalana de Recerca i Estudis Avançats (ICREA), Passeig Lluís Companys 23, 08010 Barcelona, Spain

**Keywords:** plasmonics, surface-enhanced Raman scattering, nanoparticles, biosensing, low molecular weight thiols

## Abstract

Low molecular weight thiols (biothiols) are highly active compounds extensively involved in human physiology. Their abnormal levels have been associated with multiple diseases. In recent years, major efforts have been devoted to developing new nanosensing methods for the low cost and fast quantification of this class of analytes in minimally pre-treated samples. Herein, we present a novel strategy for engineering a highly efficient surface-enhanced Raman scattering (SERS) spectroscopy platform for the dynamic sensing of biothiols. Colloidally stable silver nanoparticles clusters equipped with a specifically designed azobenzene derivative (AzoProbe) were generated as highly SERS active substrates. In the presence of small biothiols (e.g., glutathione, GSH), breakage of the AzoProbe diazo bond causes drastic spectral changes that can be quantitatively correlated with the biothiol content with a limit of detection of ca. 5 nM for GSH. An identical response was observed for other low molecular weight thiols, while larger macromolecules with free thiol groups (e.g., bovine serum albumin) do not produce distinguishable spectral alterations. This indicates the suitability of the SERS sensing platform for the selective quantification of small biothiols.

## 1. Introduction

Low molecular weight thiols play a key role in human physiology, most notably in the maintenance of cellular redox homeostasis [1]. Abnormal levels, such as those of the most abundant biothiols (e.g., glutathione (GSH) and cysteine (Cys)), have been associated with cancer [2], neurodegenerative disorders [3], and cardiovascular diseases [4,5], among others. Moreover, the overall dysregulation of the dynamic thiol-disulfide homeostasis has also been related to multiple diseases [6,7]. Due to the clinical relevance of biothiols in human health, the development of methods for their rapid determination in biological fluids is essential for early diagnosis and disease monitoring, as well as for acquiring a better understanding of biothiol-related pathophysiological processes [6,7]. It is to note that the concentrations of these biomolecules vary widely in different bodily fluids. For instance, GSH typically exists in the 1–5 μM range in plasma or serum samples from healthy human subjects, while in the whole blood, its content increases to millimolar levels [8]. 

Conventional approaches, such as those based on high-performance liquid chromatography (HPLC), capillary electrophoresis, and mass spectrometry (MS), provide robust and highly sensitive responses. However, these techniques are time-consuming, expensive, and not suitable to be used in remote settings [9]. Notably, as biothiols are prone to oxidation, methods that allow for fast quantification with minimal sample pre-treatment and no storage are particularly needed to improve the reliability of the analysis [10]. 

In recent years, major efforts have been devoted to developing new analytical approaches that would overcome the intrinsic limitations of traditional techniques, in particular fluorescent methods [9,11,12]. Several other nanosensors have also been designed to assay biothiols in biofluids beyond the realm of fluorescence, such as those based on surface-enhanced Raman scattering (SERS) spectroscopy [10,13,14,15,16,17,18,19,20,21].

SERS combines Raman spectroscopy with nanotechnology into an ultrasensitive and highly specific analytical tool. This is achieved by exploiting the giant intensification of the Raman signal from molecular scatterers located close to nanostructured plasmonic surfaces when localized surface plasmon resonances (LSPRs) are efficiently excited [22]. Consequently, SERS has been continuously integrated into a very broad range of nanosensing applications, including biomedicine and clinical diagnosis [23,24,25,26], environmental analysis [27,28,29], forensic science [30], and food safety [31]. SERS biosensing of small thiols in bodily fluids has been implemented using both direct and indirect approaches. Direct SERS detection of thiolated molecules is facilitated by the strong affinity of the mercapto group for metallic gold and silver [10,14]. Nonetheless, such an approach suffers from intrinsic limitations posed by the direct nature of the method. In particular, the competition of other molecules present in complex matrices for adsorbing onto the plasmonic surfaces can negatively impact both the robustness of the response and the discrimination of the spectral features of the biothiol from those of unknown interferences. This is further aggravated by the typical low Raman cross-section of this class of analytes. To tackle this particular issue, indirect approaches have been developed using external probes with high Raman cross-section. For instance, SERS probes binding the metallic surface via pyridinic nitrogen can be displaced by biothiols, leading to a decrease in the overall intensity (i.e., competitive adsorption) [15,16]. Alternatively, several studies took advantage of the disulfide bond-sulfide exchange reaction between GSH and Ellman’s reagent 5,5′-ditho-bis (2-nitrobenzoic acid) (DTNB) or structurally analogous molecules [17,18,19,20] to design a “SERS on” response to the presence of the biothiol. In this context, the breakage of the DTNB disulfide bridge causes the release of the two thionitrobenzoate halves, which then can covalently bind the metallic surface via their available mercapto group, giving rise to intense SERS signals. Nonetheless, these strategies do not address the limitations associated with competitive adsorptions in complex biological matrices. Recently, Shen et al. [32] developed a background-free SERS method to eliminate spurious interferences by forming a compact mixed layer of thiolated polyethyleneglycol (PEG-SH) and oligonucleotides modified by a disulfide bond. Pegylated coating prevents nanoparticle aggregation while oligo sequences act as recognition elements for biothiols. In this case, the breakage of the disulfide bond causes the release of a short DNA chain labeled with Cy5 as the SERS reporter. These nanoparticles were successfully employed for the in situ imaging and quantitative monitoring of the level of small-molecule thiols in cells. However, the manufacturing cost of oligonucleotides, especially the chemically modified ones, remains extremely high. The cost aspect is frequently ignored at the academic level but does pose a major obstacle to the successful translation of SERS into routine real-life applications [33].

In the realm of SERS-based sensing, it has been gaining great interest the design of molecular SERS transducers that can firmly bind the plasmonic surface while simultaneously presenting the ability to interact with analytes selectively. Such interactions generate target-dependent alterations of their spectral profile, which, in turn, can be quantitatively correlated with the number of binding events [34,35,36,37,38]. In this scenario, the molecular transducer (also referred to as “chemoreceptor”) also performs the role of internal standard, further boosting the robustness and reproducibility of the nanosensor response as compared to read-out strategies that solely rely on absolute intensity measurements.

With this in mind, we synthesized a mercapto-azobenzene probe (AzoProbe) to be used as an efficient and low-cost chemoreceptor in the detection of biothiols. Our choice was based on previous studies indicating that azobenzene and its derivatives undergo significant structural changes upon reduction by thiols, a property that has also been exploited for modulating the ratio between reduced and oxidized glutathione in cells [39]. The in situ generated chemoreceptor is directly conjugated to silver nanoparticles (AgNPs) with no need for tedious post-purification processes, yielding highly SERS active AgNP clusters. These aggregates are further encapsulated by PEG-SH to afford high colloidal stability in complex biological media. The SERS performances of the active substrates were tested against the clinically relevant GSH as a representative small thiolated biomolecule, demonstrating a linear range of response in the ca. 7–100 nM with an excellent limit of detection of ca. 5 nM. Thus, the sensing performance of the SERS platform fulfills the requirements for the determination of biothiols in multiple biological media. Here, the linear range for accurate quantification can be easily adjusted to the different biothiol levels (approximately from mM to μM), according to the nature of the biofluid, via mere dilution of the sample and/or by tuning the final content of SERS active clusters in the media. Moreover, this simple and low-cost nanofabrication approach drastically reduces the manufacturing cost of the SERS sensor.

## 2. Materials and Methods

### 2.1. Materials

Silver nitrate (99.8%, AgNO_3_), magnesium sulfate (99%, MgSO_4_), trisodium citrate dihydrate (≥99.5%, C_6_H_5_Na_3_O_7_·2H_2_O), and ascorbic acid (99%, C_6_H_8_O_6_), were acquired from Acros Organics. 4-aminothiophenol (≥97%, ATP), sodium nitrite (98%, NaNO_2_), and hydrochloric acid (36.5–38%, HCl) were purchased from Alfa Aesar. Phenol (99%, C_6_H_5_OH), sodium hydroxide (≥99.5%, NaOH), L-Glutathione reduced (99.72%, C_10_H_17_N_3_O_6_S), L-Glutathione oxidized (99.72%, C_20_H_32_N_6_O_12_S_2_), and poly(ethylene glycol) methyl ether thiol (99%, CH_3_O(CH_2_CH_2_O)_n_CH_2_CH_2_SH, 800 ethylene monomers repetitions, M_w_ ~ 35 kDa), were purchased from Sigma-Aldrich. Phosphate buffered saline tablets, and L-Cysteine hydrochloride monohydrate (98.5%, C_3_H_10_ClNO_3_S) were purchased from Thermo Fisher. All reactants were used without further purification. Milli-Q water (18 MU cm^−1^) was used in all aqueous solutions. All glassware was cleaned with aqua regia before the experiments.

### 2.2. Synthesis of Spherical Silver Nanoparticles (Ag NPs)

Synthesis of silver colloids was carried out as previously reported [40]. A mixture containing AA (100 μL, 0.1 M) and C_6_H_5_Na_3_O_7_·2H_2_O (1.36 mL, 0.1 M) was added under vigorous stirring to 100 mL of boiling water. After 1 min, a mixture containing AgNO_3_ (297.6 μL, 0.1 M) and MgSO_4_ (223.6 μL, 0.1 M), previously incubated for 5 min, was also added. The mixture was left to boil under stirring for 1 h. The obtained colloids were washed once via centrifugation (4000 rpm, 10 min) to remove the excess citrate and redispersed in Milli-Q water to an estimated concentration of [Ag^0^] = 3 × 10^−4^ M.

### 2.3. Synthesis of the AzoProbe

Synthesis of the AzoProbe was performed based on the report by Kar et al. [41]. Diazonium salt is first obtained by the diazotization of 4-ATP with nitrous acid. Briefly, 0.5 g of 4-ATP (4 mmol) were dissolved in 10 mL of HCl 0.001 M. Then 0.27 g of NaNO_2_ were added to the reaction. The mixture was left under gently stirring for 45 min. An ice bath was used to reach a reaction temperature below 5 °C. The colour of the mixture changes from transparent to slightly yellow. After the complete diazotization, the intermediate is coupled with C_6_H_5_OH. A total of 0.46 g (ca. 4.8 mmol) of the latter were dissolved in NaOH (55.7 μL, 0.5 M), cooled, and slowly added to the mixture. The reaction is left under mild stirring for 45 min at a temperature below 5 °C. The final solution (AzoProbe reaction mixture) turns from slightly yellow to gold. 

### 2.4. Functionalization of the Ag Nanoparticles with the AzoProbe (Ag@AzoProbe) and Polyethylene Glycol (PEG) Encapsulation (Ag@AzoProbe@PEG)

2 mL of the AgNPs colloids were added dropwise under vigorous stirring to 0.8 mL of AzoProbe reaction mixture. The obtained mixture was left to incubate overnight with orbital shaking and then centrifuged once (1000 rpm, 10 min). The pellet was resuspended in 2 mL of Milli-Q water and combined with 530 μL of poly(ethylene glycol) methyl ether thiol (PEG-SH) 10^−6^ M under vigorous stirring [42]. The solution was stirred overnight. The excess of the polymer was removed via centrifugation (1000 rpm, 10 min), and the encapsulated aggregates Ag@AzoProbe@PEG were collected as pellets and redispersed in 100 μL phosphate buffer saline (PBS, pH 7.4). The amount of PEG-SH was calculated to match the one corresponding to a full monolayer coverage of the silver nanoparticles considering a polymer footprint of 2.18 nm^2^ (2.65 × 10^−10^ mol of PEG-SH per 1 mL of Ag colloids containing ca. 3 × 10^10^ NPs) [42].

Samples for SERS analysis were prepared by adding 10 μL of Ag@AzoProbe@PEG suspension to 200 μL of GSH solution in PBS (pH 7.4) at different concentrations. The sample was incubated 1 h before the measurement. An identical protocol was applied for other low molecular weight biothiols (glutathione disulfide, GSSG; and cysteine, Cys) and high molecular weight biothiols (bovine serum albumin, BSA). Silver nanoparticles previously incubated with 4-aminothiophenol (4-ATP) 10^−7^ M were aggregated by adding 30 μL of a 0.5 M solution of MgSO_4_ to 1 mL of colloids to acquire an intense SERS fingerprint of 4-ATP on AgNPs. 

### 2.5. Instrumentation

UV-VIS spectroscopy (Thermo Scientific Evolution 201) and transmission electron microscopy (TEM, JEOL 1011 operating at 100 kV) were used to characterize the optical response and size of the nanoparticles. TEM samples were prepared by drying water suspensions on carbon-Formvar-coated 200 mesh copper grids. SERS spectra were collected in backscattering geometry with a Renishaw inVia Reflex system equipped with a 2D-CCD detector, a Leica confocal microscope, and a 785 nm laser line. The laser was focused on the colloidal suspension using a macrolens (power at the sample = 3 mW, 1 s accumulation).

## 3. Results and Discussion

**Figure 1:**
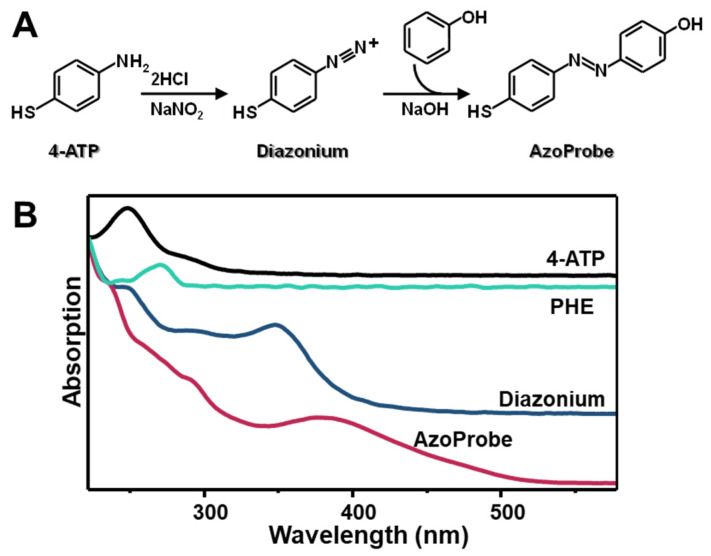
(**A**) Outline of the synthetic route for mercaptophenyl azo phenol (AzoProbe) preparation. (**B**) UV-Vis spectra of the precursors 4-aminothiophenol (4-ATP) and phenol (PHE), the in situ generated diazonium cation and the AzoProbe reaction mixture.

Structural requirements of SERS chemosensors commonly involve the presence of a mercapto group to firmly anchor the metallic surface [36,37,43,44]. Thus, we designed an azobenzene derivative by combining 4-aminothiophenol (4-ATP) and phenol (PHE) (Figure 1A). First, sodium nitrite and hydrochloric acid were combined to generate nitrous acid in situ, which, in turn, reacts with 4-ATP to yield the corresponding diazonium cation. Then, upon addition of phenol, the azo-coupling reaction takes place, leading to the formation of the corresponding mercaptophenyl-azo-phenol (AzoProbe) via electrophilic aromatic substitution [41]. Figure 1B illustrates the corresponding UV-Vis spectra at each stage of the process, confirming the successful generation of the AzoProbe. It is to be noted that, in our case, a slight excess of PHE reactant was employed to maximize the 4-ATP consumption. In this way, the reaction mixture can be directly combined with silver nanoparticles without tedious post-purification steps since all chemicals, barring AzoProbe, do not bind the metal surface and can be easily removed from the medium in subsequent washing cycles.

**Figure 2:**
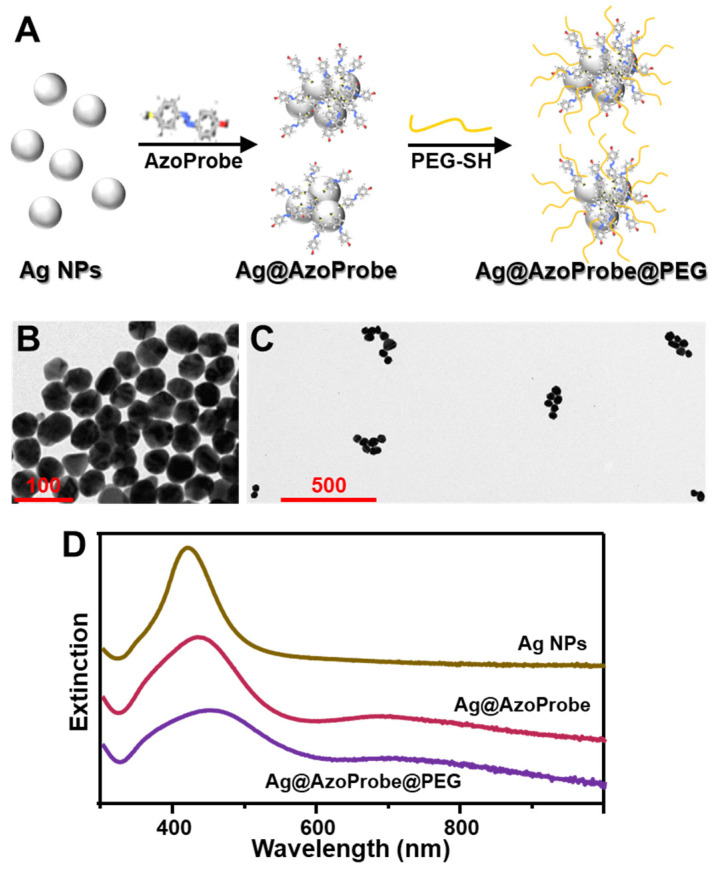
(**A**) Outline of the fabrication of colloidally stable silver nanoparticle clusters modified with the AzoProbe molecular sensor and thiolated PEG. (**B**,**C**) Representative TEM images of AgNPs and Ag@AzoProbe@PEG clusters, respectively (scale bars = 100 and 500 nm). In the case of Ag@AzoProbe@PEG clusters, the sample has been highly diluted prior deposition to minimize nanoparticle aggregation induced by the drying process. (**D**) Extinction spectra of silver colloids before (Ag NPs) and after (Ag@AzoProbe) the addition of the azo-compound in Milli-Q water, and the corresponding pegylated clusters (Ag@AzoProbe@PEG) in PBS buffer (pH 7.4).

The methodological approach for SERS substrate fabrications is outlined in Figure 2A. Silver colloids (AgNPs) of ca. 55 ± 5 nm diameter (Figure 2B) were prepared via the conventional chemical reduction method using sodium citrate [40]. An aliquot of AgNPs was combined with the AzoProbe reaction mixture to a final probe concentration of ca. 0.1 mM. Such AzoProbe content is estimated to be sufficiently high to promote the formation of a full monolayer via direct covalent binding with the pending SH group of the chemosensor. The sample was incubated overnight to maximize the AzoProbe surface density on Ag colloids. Such chemical modification causes the localized surface plasmon resonance (LSRP) of silver colloids, initially centered at 421 nm, to undergo a red-shift to ca. 438 nm and a marked broadening (Figure 2D). Moreover, a new contribution emerges at ca. 700 nm, indicating the formation of metastable nanoparticle aggregates (Ag@AzoProbe) in Milli-Q water. 

In order to enhance the colloidal stability of the Ag@AzoProbe in complex media, PEG-SH (35 kDa) was successively added to the colloidal mixture to yield Ag@AzoProbe@PEG clusters [42]. PEG-SH is a neutral, water-soluble, and biocompatible polymer that has been extensively exploited as a surface stabilizer to protect metallic nanoparticles from aggregation against salts [45]. After overnight incubation, the colloidal dispersion was submitted to low-speed centrifugation to remove unbound polymer molecules. The pellet was collected and redispersed in PBS (pH = 7.4), while the light-yellow supernatant was discarded. In this way, the residual fraction of individual, non-clustered nanoparticles in the supernatant was largely removed from the medium, as it can be also inferred from the weakening of the plasmonic contribution at 421 nm in the extinction spectra (Figure 2D). This enabled the separation of highly SERS active Ag@AzoProbe@PEG (Figure 2C) clusters from poorly efficient enhancers. On the other hand, non-pegylated clusters redispersed in PBS buffer underwent a slow but irreversible aggregation over time.

**Figure 3:**
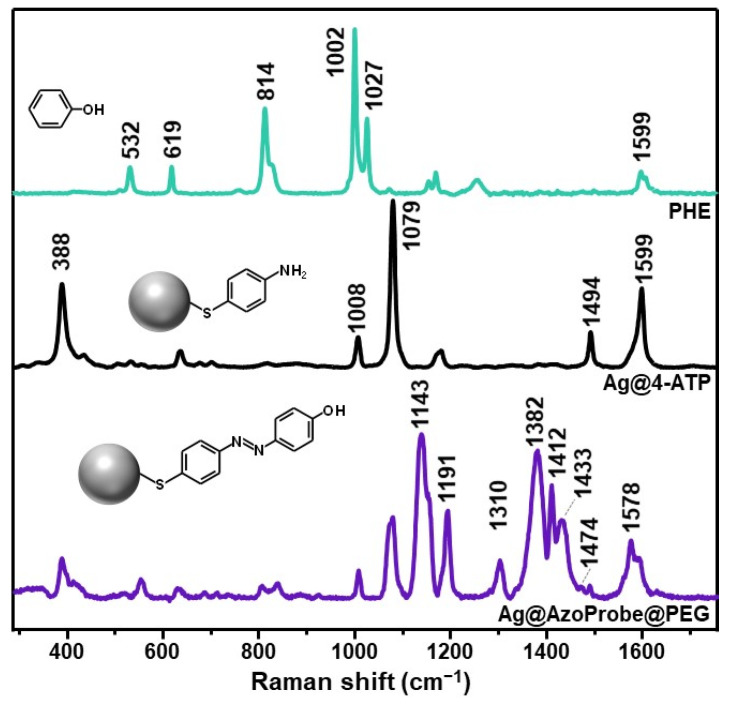
Normal Raman spectrum of phenol (PHE) and SERS spectra of 4-ATP on aggregated AgNPs and AzoProbe in Ag@AzoProbe@PEG clusters.

Figure 3 compares the SERS spectrum of Ag@AzoProbe@PEG clusters with that of silver colloids modified with the AzoProbe precursor 4-aminothiophenol (Ag@4-ATP). Differently from 4-ATP, PHE does not show any affinity for the Ag surface and thus, the normal Raman spectrum of the solid phenol was acquired instead. Neighboring 4-ATP molecules, however, undergo photocatalyzed diazotization onto silver nanostructures to yield the corresponding 4,4′-dimercaptoazobenzene (DMAB) product [29,46]. To avoid dimerization, 4-ATP was then added at a low concentration (10^−7^ M) to the AgNPs suspension [29]. The so-functionalized nanoparticles were then aggregated using a MgSO_4_ solution as the aggregating agent to yield a detectable SERS spectrum of the non-dimerized molecule (Figure 3, Ag@4-ATP). The most intense 4-ATP bands are centered at 368, 1008, 1079, 1494, and 1599 cm^−1^, which have been ascribed to out-of-plane C-C-C modes (368 and 1008 cm^−1^), ring breathing and C-S stretching (1079 cm^−1^), N-H bending (1494), and C=C stretching (1599 cm^−1^) [47]. On the other hand, the SERS spectrum of the synthesized AzoProbe (Figure 3, Ag@AzoProbe@PEG) displays new intense features which are informative of the formation of the -N=N- bond between the 4-ATP and PHE precursors. Most notably, we highlight the bands at 1143, 1191, 1310, 1382, 1433, and 1578 cm^−1,^ which can be mainly assigned to a combination of N-C stretching and C-H bending (1143, 1191, and 1433 cm^−1^), N-C and C-H bending (1310 cm^−1^), and C-C and N-N stretching (1382 and 1578 cm^−1^) [47]. Furthermore, the intense band at 1412 cm^−1^ and the weak feature at 1474 cm^−1^ have been previously assigned to the *trans* and *cis* forms of the analogous structural 4-phenylazophenol, respectively [48].

**Figure 4:**
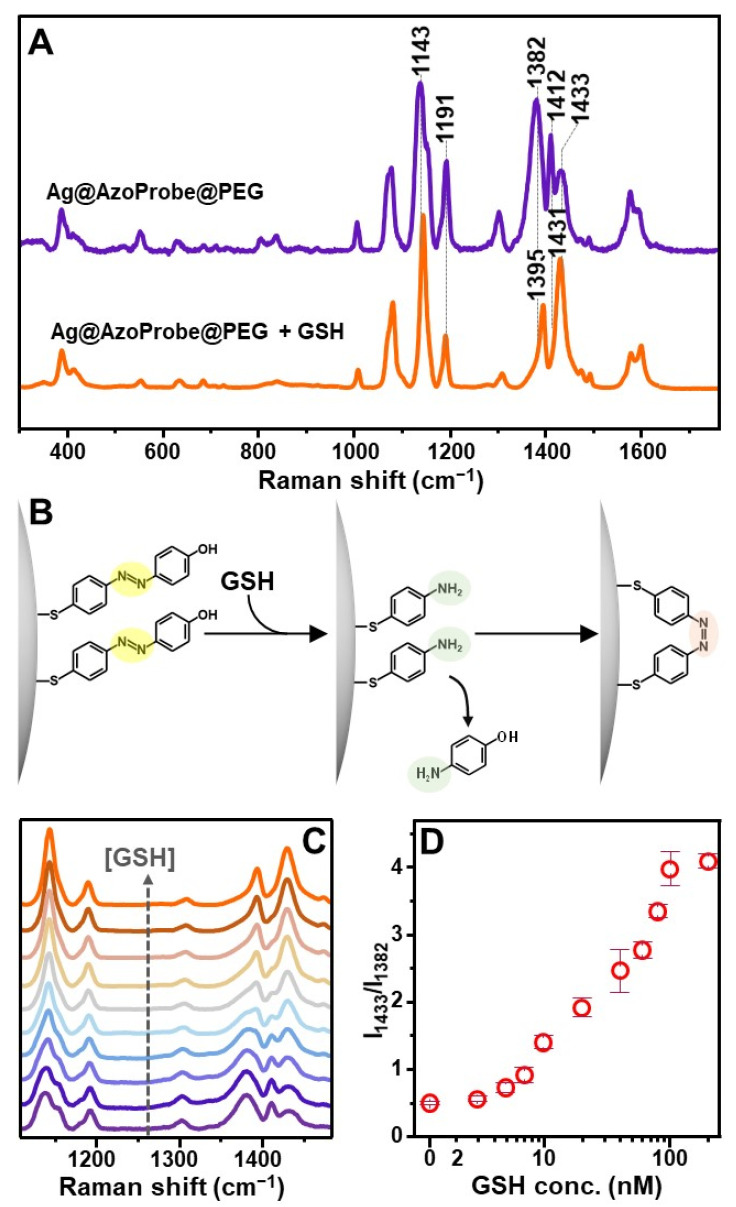
(**A**) SERS spectra of Ag@AzoProbe@PEG clusters before and after mixing with GSH (100 nM). (**B**) Outline of the proposed detection mechanism. (**C**) From bottom to top, SERS spectra of Ag@AzoProbe@PEG clusters at increasing GSH concentration (0, 5, 7, 10, 20, 40, 60, 80, 100, and 200 nM). (**D**) Intensity ratio I_1433_/I_1382_ vs. GSH concentration (logarithmic scale).

As a representative small thiolated biomolecule, we selected the clinically relevant GSH, which is also the most abundant intracellular nonprotein thiol [49]. The SERS response of the Ag@AzoProbe@PEG material to the presence of GSH was monitored in samples obtained by diluting 10 μL of highly SERS-active clusters into 200 μL of GSH solution in PBS buffer (pH = 7.4). Figure 4A shows the SERS spectra of Ag@AzoProbe@PEG before and after the addition to a 100 nM solution of GSH (spectra were collected after 1 h incubation at room temperature). The substrate exposure to glutathione leads to a major reshaping of the molecular probe spectrum, demonstrating the ability of GSH to diffuse across the external PEG shell and onto the AzoProbe functionalized metallic surface. Most notably, spectral reshaping involves those bands associated with vibrations of the azo bond that links the two aromatic moieties (see 1110–1520 cm^−1^ spectral range). As it has been reported, in response to GSH, azo dyes undergo reductive cleavage to yield the respective amines [50]. In our scenario, this implies the release of the 4-aminophenol moiety from the silver surface while the residual 4-ATP fragment remains anchored via the strong Ag-S bond (Figure 4B). However, in this case, dense patches of closely spaced 4-ATP molecules are left behind on Ag surfaces, including at the interparticle gaps, which then triggers their surface catalyzed dimerization upon laser illumination (Figure 4B). Indeed, the SERS spectrum of the GSH-treated clusters matches that of the self-diazotization product DMAB on silver colloids [29,46]. 

The evolution of the SERS profile in the spectral range of interest (1110–1520 cm^−1^) as a function of a decreasing GSH concentration (from 100 nM to 3 nM) is illustrated in Figure 4C. To correlate the spectral reshaping with the analyte content, we calculated the SERS intensity ratio between the 1433 and 1382 cm^−1^ bands (I_1433_/I_1382_), which has been selected as the spectral marker due to its extensive variation in the presence of GSH. The resulting intensity ratios are plotted against GSH concentration in Figure 4D, showing a good linear relation in the 7–100 nM range (r^2^ = 0.978) and an estimated limit of detection of ca. 5 nM. 

It is worth stressing that all SERS experiments were performed by focusing a 785 nm laser onto the colloidal samples using a macrolens. In this manner, we obtain statistically averaged SERS spectra from a relatively high number of clusters in continuous Brownian motions within the scattering volume. At the same time, the Ag@AzoProbe@PEG clusters concentration in the sample was maintained sufficiently high to yield intense SERS spectra with well-defined features adopting an integration time of just 10 s. The intrinsically high sensitivity of the proposed method allows obtaining detection limits that far exceed those required to detect biothiols at biologically relevant concentrations in bodily fluids. Thus, we were able to adopt an experimental set-up that favors rapidity, simplicity, and spectral reproducibility, as required for quantitative applications [51], although at the expense of the absolute detection sensitivity. 

**Figure 5:**
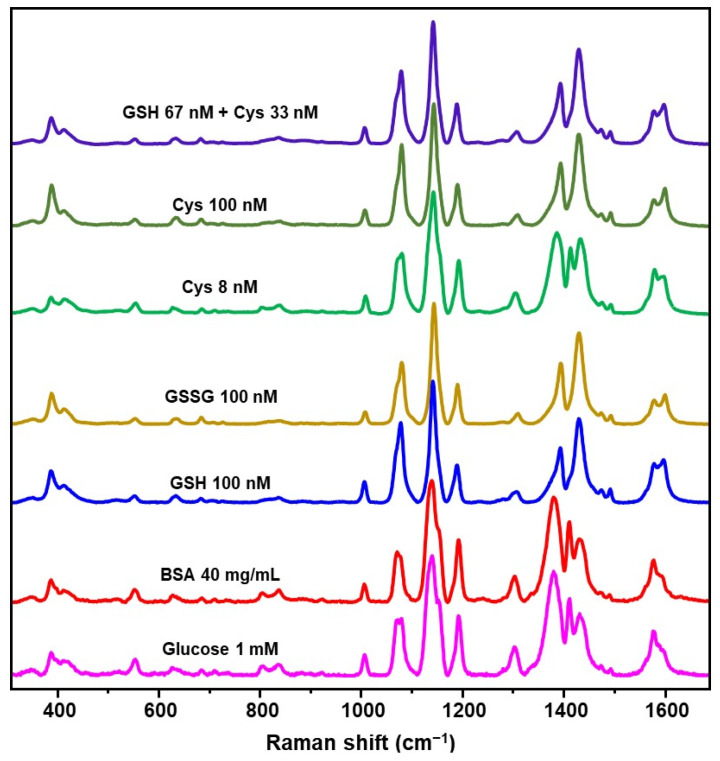
SERS spectra of Ag@AzoProbe@PEG clusters in PBS buffer (pH 7.4) containing glucose (1 mM), BSA (40 mg/mL), GSH 100 nM, glutathione disulfide (GSSG) 100 nM, cysteine (Cys) 8 and 100 nM, and a mixture of GSH+Cys (6.7 × 10^−8^ M and 3.3 × 10^−8^ M, respectively).

Identical spectral changes as those observed in Figure 4 for GSH (100 nM) have also been recorded for other small biothiols such as oxidized glutathione (GSSG) and cysteine (Cys) at the same concentration (Figure 5). A similar outcome emerged when the nanoprobes were exposed to a mixture of GSH and Cys at a 2:1 molar ratio (total biothiol content = 100 nM). Notably, when the Cys content is decreased to 8 nM, the intensity ratio I_1433_/I_1382_ value (ca. 0.95) approaches that of GSH (ca. 1.0) at an analogous concentration. As also previously reported [21], thiolated PEG coating of nanoparticles allows for small biothiols to approach the metallic surface while blocking large macromolecules. In order to verify such quality of Ag@AzoProbe@PEG clusters, we exposed the nanostructures to a 40 mg/mL solution of bovine serum albumin (BSA), which was selected as a representative example of biomacromolecule equipped with thiol groups. No changes in the AzoProbe spectrum (Figure 5) were detected, which confirms the impossibility of large proteins to diffuse at the interparticle gaps within the clusters. Indeed, as it has been extensively demonstrated in the literature, the SERS signal from aggregates composed of closely-spaced nanoparticles is dominated by the contribution of the fraction of molecules that are entrapped at the interparticle gaps [52,53]. We also tested the response of the sensing platform in the presence of 1 mM glucose, which was chosen as a representative non-thiolated and ubiquitous reducing chemical in biofluids. No alterations of the AzoProbe SERS spectrum were detected (Figure 5). On the other hand, small molecules such as ascorbic acid and hydroxyl radicals, that showed heterogeneous reactivity with azo groups [54,55], coexist with low molecular weight thiols in biofluids at concentrations well below the overall biothiol content, a condition that provides the basis for discrimination.

In summary, we have reported a novel approach for engineering a highly efficient, low-cost platform for the dynamic SERS sensing of biothiols. An azobenzene derivative equipped with a mercapto group has been designed to firmly bind silver colloids via Ag-S bond while generating metastable and highly SERS active clusters in suspension. Subsequent encapsulation of the so-formed clusters with PEG-SH granted the required colloidal stability to be used in complex media such as biofluids. When exposed to GSH, the SERS spectrum of the AzoProbe displays major spectral changes, which were ascribed to the breakage of the diazo bond, causing the release of the 4-aminophenol moiety from the surface. The residual surface-bound 4-aminothiophenol fragment is then free to undergo metal-catalyzed dimerization with other neighboring molecules. The extent of such spectral alterations was quantitatively correlated with the GSH content in the ca. 7–100 nM range with an excellent limit of detection of ca. 5 nM. It is worth stressing that the linear range for accurate quantification can be simply tuned to different biothiol concentration ranges by merely varying the absolute content of SERS active clusters or via sample dilution. An identical response was observed for other low molecular weight thiols (i.e., oxidized glutathione and cysteine), while larger macromolecules with free thiol groups such as BSA do not produce distinguishable spectral alterations, indicating the suitability of the SERS sensing platform for the selective quantification of small biothiols.

## Data Availability

The data presented in this study are available on request from the corresponding authors.
